# Re: Barriers and facilitators to adoption of soft copy interpretation from the user perspective: Lessons learned from filmless radiology for slideless pathology. J Pathol Inform, 2011;2:1, Patterson *et al*

**DOI:** 10.4103/2153-3539.77170

**Published:** 2011-02-26

**Authors:** Andrew J. Evans

**Affiliations:** Department of Pathology, University Health Network, Toronto General Hospital, Toronto, ON, Canada

There are definite lessons to be learned from the digital radiology experience as pathology transitions toward more widespread use of digital images for diagnostic purposes. While there are parallels between digital radiology and digital pathology in terms of work flow gains and losses, there are also important differences like pathology’s need for color images and the large files that are created when slides are digitized as whole slide images (WSI). The paper by Patterson *et al*,[1] points out that the adoption of digital imaging by pathologists has been slower than that encountered with filmless radiology. To explore this issue deeper, the authors conducted semi-structured interviews with radiologists and pathologists and looked at the adoption of other health information technologies. Their results indicated that pathologists have a lower opinion of the overall performance of digital systems than their radiologist counterparts. Specific issues of concern included: differences in magnification and image scale as compared to light microscopy, large file sizes and data management, longer time to review individual slides and an inability to focus on folded or uneven areas of tissue. In addition, hardware and software costs, information technology (IT) support, LIS integration, regulatory issues and lack of standards or best practice guidelines also represent key obstacles to the more widespread adoption of digital pathology.

Even if the issues around regulations, standards, cost and infrastructure were to suddenly vanish, the perception of inferior performance of WSI systems by pathologists will prevent more rapid adoption. The issue of inability to adjust focus on digital images on folded or uneven areas of tissue highlights the dependence of current-state digital pathology systems on good quality histology. It also raises obvious concerns about diagnostic accuracy. As pointed out by one of the pathologists in the study by Patterson *et al*, inferior performance (assuming this refers to diagnostic accuracy) on even a small percentage of cases could have major implications for high risk diagnoses. The importance of this point as a barrier to adoption cannot be overemphasized. As pointed out in a recent *Scientific American* article,[2] the practice of pathology has been based on glass slides and light microscopes for over 100 years and digital pathology systems represent disruptive technology. The prospect of such a major change will naturally cause pathologists to be reluctant about adopting a technology that could both slow them down and introduce the possibility of diagnostic error. Having said this, it must also be acknowledged that WSI technology is steadily improving and vendors are acutely aware of the need for outstanding image quality and faster scanning speeds. Validation studies performed in a variety of institutions and settings using these improved systems will play a critical role in determining the rate of adoption. Whether these studies are based on histologic feature recognition, diagnostic concordance or a combination of the two, the results must demonstrate equivalence between WSI systems and light microscopy if a sense of confidence is to develop across the pathology community as a whole.

The survey by Patterson *et al*, identified many facilitators for the adoption of digital pathology. These included the benefits of using digital pathology platforms for medical student and pathology resident training, continuing medical education and tumor boards. All of these activities share a theme of increasing the exposure of the pathologists to this technology using optimized images and presenting them in an environment that is essentially free of worry over diagnostic accuracy. Such exposure should only be beneficial in terms of building a comfort level among pathologists, especially as the technology continues to improve and new generations of pathologists encounter WSI technology throughout their residency training.

The paper by Patterson *et al*, also includes a comprehensive list of survey questions that was pilot tested at a recent pathology informatics conference. The list of questions explores issues related to work environment in digital pathology as compared to light microscopy. I feel that this is an important and relatively underexplored aspect of digital pathology. It may be that pathologists will need to adjust their work environment so as to minimize the chance of being distracted by incoming e-mail or being interrupted by others while trying to sort out a live frozen section on their computer screen. As a pathologist who currently uses WSI to read frozen sections at my institution,[3] it has been my experience that I am significantly (dare I say without implying rigorous data collection with supporting statistics!) more likely to be interrupted when looking at cases on my office computer screen as opposed to using my microscope.

While a lot of work remains to be done before pathology reaches the level of adoption seen in digital radiology, I agree with the comment by Patterson *et al*, the performance barriers are tractable. The big question is how long the process will take. Once the performance problems have been overcome, the full potential of WSI systems as a basis for diagnostic telepathology networks can be realized in terms of improving access to sub-specialty diagnostic opinions and providing pathology services to remote or underserviced locations.
